# Genetic Parameters of White Striping and Meat Quality Traits Indicative of Pale, Soft, Exudative Meat in Turkeys (*Meleagris gallopavo*)

**DOI:** 10.3389/fgene.2022.842584

**Published:** 2022-03-03

**Authors:** Ryley J. Vanderhout, Emily M. Leishman, Emhimad A. Abdalla, Shai Barbut, Benjamin J. Wood, Christine F. Baes

**Affiliations:** ^1^ Centre for Genetic Improvement of Livestock, University of Guelph, Guelph, ON, Canada; ^2^ Department of Food Science, University of Guelph, Guelph, ON, Canada; ^3^ Hybrid Turkeys, Kitchener, ON, Canada; ^4^ School of Veterinary Science, University of Queensland, Gatton, QLD, Australia; ^5^ Institute of Genetics, Vetsuisse Faculty, University of Bern, Bern, Switzerland

**Keywords:** breast meat, correlation, heritability, meat quality, poultry

## Abstract

Due to the increasing prevalence of growth-related myopathies and abnormalities in turkey meat, the ability to include meat quality traits in poultry breeding strategies is an issue of key importance. In the present study, genetic parameters for meat quality traits and their correlations with body weight and meat yield were estimated using a population of purebred male turkeys. Information on live body, breast, thigh, and drum weights, breast meat yield, feed conversion ratio, breast lightness (L*), redness (a*), and yellowness (b*), ultimate pH, and white striping (WS) severity score were collected on 11,986 toms from three purebred genetic lines. Heritability and genetic and partial phenotypic correlations were estimated for each trait using an animal model with genetic line, hatch week-year, and age at slaughter included as fixed effects. Heritability of ultimate pH was estimated to be 0.34 ± 0.05 and a range of 0.20 ± 0.02 to 0.23 ± 0.02 for breast meat colour (L*, a*, and b*). White striping was also estimated to be moderately heritable at 0.15 ± 0.02. Unfavorable genetic correlations were observed between body weight and meat quality traits as well as white striping, indicating that selection for increased body weight and meat yield may decrease pH and increase the incidence of pale meat with more severe white striping. The results of this analysis provide insight into the effect of current selection strategies on meat quality and emphasize the need to include meat quality traits into future selection indexes for turkeys.

## Introduction

With an increasing desire for lean, quality poultry meat products emphasis needs to be placed on improving technological characteristics and functional properties of the meat (i.e., pH, colour, water-holding capacity) (Barbut, 2015; Petracci et al., 2015). However, it is suggested that intense selection for growth and yield in poultry could be associated with a greater occurrence of growth-related myopathies and abnormalities, consequently increasing the number of downgraded carcasses and leading to an overall reduction of meat quality (Sosnicki and Wilson, 1991; Updike et al., 2005; Owens et al., 2009; Zampiga et al., 2020). The rate and magnitude of postmortem decline in muscle pH greatly affects the overall quality of the final meat product (Briskey, 1964; Wynveen et al., 1999; Barbut et al., 2008). Differences in postmortem muscle pH among individual birds arise due to changes in rates of glycolysis which can be affected by several factors including pre-slaughter handling (stress), stunning method, muscle size, carcass chilling, nutrition, and genetics (Ma et al., 1971; Rathgeber et al., 1999; Wynveen et al., 1999; Velarde et al., 2000). When there is a rapid decline in pH or an exceptionally low pH in the final meat product, the result is pale, soft, exudative (PSE) meat. Characteristics of PSE meat include increased functional protein degradation leading to lighter coloured meat with decreased water-holding capacity and sometimes an increased shear force of the cooked product (Barbut, 1993, 1997; Owens et al., 2002). Not only is this difference in quality visually noticeable in broiler chicken meat, but it also affects sensory acceptability, with panelists preferring cooked meat classified as normal over the PSE meat (Droval et al., 2012).

In addition to the rise in PSE meat observed in the poultry industry over the past decades, an increase in the incidence of the growth-related myopathy white striping (WS) has been observed in the turkey industry (Mudalal, 2019; Vanderhout et al., 2022). This myopathy presents itself as thin white striations on the surface of the muscle running parallel to the muscle fibers. These white striations are a result of muscle tissue necrosis and subsequent infiltration of fat and connective tissue into the muscle (Kuttappan et al., 2013b; Baldi et al., 2018; Barbut, 2019). The resulting product exhibits an increase in lipid content while decreasing myofibrillar protein content (Kuttappan et al., 2016; Soglia et al., 2018; Barbut, 2019). This not only affects the quality of further processed products but also negatively affects consumer acceptance of broiler chicken whole muscle products (Kuttappan et al., 2012; de Carvalho et al., 2020). Estimates for heritability of WS in broiler chickens range from 0.19 to 0.65 (Bailey et al., 2015; Alnahhas et al., 2016; Lake et al., 2021), however, there are no published estimates for turkeys.

Estimation of genetic parameters of meat quality traits is crucial to evaluate the possibility of genetic selection and more importantly, the magnitude of indirect selection on these traits. Due to the complexity of measuring these traits, research on their genetic parameters in turkeys is limited in comparison to other species and when conducted, generally have small sample sizes (Le Bihan-Duval et al., 2003; Aslam et al., 2011). However, the initial published estimates do show low to moderate heritabilities for breast pH and colour along with moderate to strong unfavorable genetic correlations among these traits and body weight (Le Bihan-Duval et al., 2003; Aslam et al., 2010). Therefore, the objectives of this study were to measure WS and key meat quality traits indicative of PSE meat in a large turkey population and to estimate genetic parameters for these traits. Additionally, we determined their phenotypic and genetic correlations with key economic traits such as growth and feed efficiency.

## Materials and Methods

### Animals

Data were collected on male turkeys from three purebred genetic lines (A, B, and C) over 44 weeks between July 2018 and November 2019. There were 11,986 birds included in this study; 2,569 were from line A, 5,299 from line B, and 4,118 from line C. The genetic lines included a dam-line that was selected primarily for body weight and reproductive traits (line A), a dam-line selected mainly for reproductive traits (line B), and a sire-line with selection focused on body weight, meat yield, and feed efficiency (line C). All genetic lines were raised under the same husbandry conditions (Hybrid Turkeys, 2020). Processing occurred between 20–24 weeks of age (average body weight of 21.5 kg) at a commercial poultry processing facility in Ontario, Canada. During processing, birds were electrically stunned and exsanguinated. Birds were scalded, defeathered, and eviscerated before moving to the chiller for 24 h prior to deboning and collecting meat quality measurements and carcass component weights.

### Production Traits

Body weight (BW) was collected 2 days prior to slaughter (20–24 weeks of age). A real-time automated system was used to record individual feed intake (Tu et al., 2011) and feed conversion ratio (FCR) was calculated as total feed intake divided by weight gain (Abdalla et al., 2019). Carcass component weights were collected approximately 24 h postmortem. Randomly selected carcasses were broken down into the major components, and *Pectoralis major* (fillets), *Pectoralis minor* (tenders), thighs (bone-in, skin on), and drums (bone-in, skin on) were individually weighed. All remaining carcasses were processed separately, and the weight of total breast meat (fillets and tenders; BrW) were measured. All weights were measured in kg. Breast meat yield (BMY) was calculated as a percentage of BW.

### Meat Quality Measurements

Meat quality measurements included ultimate pH and color. A pH measurement was taken from the dorsal side of an intact, deboned fillet from the randomly selected, broken down carcasses at 24 h post-mortem (pHu; Portable pH meter HI98163, Hanna Instruments, Woonsocket, RI, United States). Breast lightness, redness, and yellowness (L*, a*, and b*; CIE, 2018) were measured on the skinless dorsal side of the fillet of all birds using a colorimeter with D50 illumination (Nix Pro Colorimeter, Hamilton, ON, CA). All fillets were also photographed (Hero 6, GoPro, San Mateo, CA, United States) approximately 24 h post-mortem. Photographs were used to evaluate WS on a 0–3 scoring scale (Kuttappan et al., 2016; Vanderhout et al., 2022).

### Statistical Models

Univariate and bivariate linear animal models were used to estimate (co)variance components through restricted maximum likelihood carried out using the BLUPf90 family of programs (Misztal et al., 2018). The linear animal models used can be described as follows:

y=Xb+Za+e, (1)

where **y** is the vector of observations sorted within animals; **b** is a vector of fixed effects including genetic line (3 levels: A, B, and C), hatch week-year (58 levels), and age at slaughter (7 levels; 141–163 days) for all models and the addition of score observer (6 levels) for WS; **a** is a vector of additive genetic effects distributed as a ∼ N(0, A⊗G) , where **A** is the numerator relationship matrix including the inbreeding coefficients and **G** is the additive genetic variance-covariance matrix between traits; **e** is the vector of residual effects which has a distribution of $e∼N\left(0,\overset{+}{\underset{i}{\sum }}E_{iy}\right)$ where **E**_*iy*_ is an m_*i*_ x m_*i*_ matrix corresponding to the traits that were present for animal *i* and m_*i*_ is the number of traits present for animal *i*; and **X** and **Z** are design matrices relating the observations to the fixed and random effects, respectively. Heritability (h^2^) was estimated as the proportion of phenotypic variance (sum of the additive genetic variance and residual variance) explained by additive genetic variance estimated from the univariate models. Genetic correlation coefficients (**r**_**g**_) were calculated using the following equation:

rg=σxyσx2σy2, (2)

where ${\text{σ}}_{xy}$ is the additive genetic covariance of traits x and y, and ${\text{σ}}_{x}^{2}$ and ${\text{σ}}_{y}^{2}$ are the additive genetic variances for traits *x* and *y*, respectively. Pearson partial phenotypic correlation coefficients (**r**_**p**_) were calculated using PROC GLM in SAS (version 9.4, SAS Institute Inc., Cary, NC, United States) to account for the fixed effects included in the genetic (co)variance estimates.

## Results and Discussion

### Heritability Estimates

Analysis of the 13 traits (Table 1) resulted in 78 bivariate combinations. The heritability estimates (Table 2) for all traits were moderate to high (0.15–0.63). Estimates for BW (*h* ^2^ = 0.44 ± 0.03) and BMY (*h* ^2^ = 0.41 ± 0.02) were high and within the range of previously published estimates in turkeys, which ranged from 0.23 to 0.45 for BW (Le Bihan-Duval et al., 2003; Case et al., 2012a, 2012b; Willems et al., 2013; Abdalla et al., 2019) and 0.27 to 0.43 for BMY (Le Bihan-Duval et al., 2003; Aslam et al., 2011; Case et al., 2012b; Abdalla et al., 2019). Carcass component weights (i.e., fillets, tenders, thighs, and drums) all showed high heritabilities similar to those previously published in both turkeys ranging from 0.45 to 0.49 (Case et al., 2012b) and broiler chickens ranging from 0.38 to 0.61 (Felício et al., 2013; Alnahhas et al., 2016). Breast weight (BrW), fillets, and tenders had similar (co)variance estimates and therefore, the remainder of the discussion will focus on BrW. A moderate heritability estimate was observed for FCR (*h* ^2^ = 0.18 ± 0.03) which was slightly higher than previously published estimates in turkeys which ranged from 0.05 to 0.16 (Case et al., 2012a; Willems et al., 2013; Abdalla et al., 2019). The heritability estimates presented in the present study for body and carcass component weights and FCR, along with previously published estimates, emphasize the strong genetic aspect of weight and efficiency in turkeys. It is to no surprise that considerable gains have been made in these areas over time (Havenstein, 2006).

**TABLE 1:** Descriptive statistics (count, mean, and standard deviation) for the 13 recorded traits. Traits were recorded on three purebred genetic lines.

Trait	Abbreviation	Trait unit	Line A		Line B		Line C		All lines
			N	Mean (SD)	N	Mean (SD)	N	Mean (SD)	Mean (SD)
Body weight^a^	BW	kg	2,564	21.77 (1.61)	5,285	19.12 (1.35)	4,102	24.57 (1.73)	21.56 (2.85)
Breast weight	BrW	kg	2,553	5.14 (0.60)	5,215	4.74 (0.50)	4,033	6.04 (0.75)	5.27 (0.84)
Fillets	—	kg	659	4 0.47 (0.49)	1,431	4.06 (0.42)	1,011	5.13 (0.63)	4.50 (0.69)
Tenders	—	kg	660	0.83 (0.09)	1,426	0.77 (0.07)	1,009	1.01 (0.11)	0.86 (0.14)
Breast meat yield	BMY	% BW	2,550	23.61 (1.94)	5,203	24.77 (1.71)	4,024	24.52 (2.05)	24.43 (1.93)
Thighs	—	kg	667	3.00 (0.26)	1,478	2.51 (0.21)	1,072	3.44 (0.29)	2.92 (0.48)
Drums	—	kg	646	2.34 (0.20)	1,460	1.94 (0.15)	1,050	2.59 (0.21)	2.24 (0.34)
Feed conversion ratio	FCR	kg/kg	827	2.37 (0.36)	1,604	2.44 (0.33)	3,318	2.51 (0.39)	2.47 (0.37)
Lightness	L*	—	1,921	37.40 (2.54)	3,671	38.05 (2.58)	2,957	37.48 (2.68)	37.71 (2.62)
Redness	a*	—	1,921	3.23 (0.66)	3,671	3.21 (0.70)	2,957	2.94 (0.65)	1.12 (0.69)
Yellowness	b*	—	1,921	5.09 (0.91)	3,671	4.90 (0.94)	2,957	4.92 (0.93)	1.95 (0.93)
Ultimate pH	pHu	—	643	5.75 (0.12)	1,340	5.77 (0.11)	1,041	5.79 (0.11)	5.77 (0.11)
White Striping (0–3)	WS	—	1,838	2.62 (0.77)	3,728	2.61 (0.81)	2,856	2.31 (0.77)	2.51 (0.80)

a Measured 2 days prior to slaughter (20–24 weeks).

**TABLE 2:** Heritability^a^ (diagonal), additive genetic correlation coefficients^b^ (above diagonal), and partial phenotypic correlation coefficients (below diagonal).

	BW	BrW	Fillets	Tenders	BMY	Thighs	Drums	FCR	L*	a*	b*	pHu	WS
BW	**0.44**	0.74	0.73	0.49	0.18	0.73	0.66	−0.09	0.31	0.10	0.16	−0.18	0.26
BrW	0.78^c^	**0.46**	0.99	0.53	0.79	0.33	0.24	−0.03^d^	0.43	0.06^d^	0.19	−0.19	0.25
Fillets	0.76^c^	0.99^c^	**0.47**	0.45	0.78	0.37	0.27	0.16	0.38	0.08^d^	0.16	−0.22	0.42
Tenders	0.52^c^	0.61^c^	0.48^c^	**0.50**	0.32	0.48	0.41	0.03^d^	0.13	−0.09	0.17	−0.01^d^	−0.08^d^
BMY	0.29^c^	0.83^c^	0.82^c^	0.47^c^	**0.41**	−0.23	−0.27	0.04^d^	0.37	0.00^d^	0.14	−0.09	0.13
Thighs	0.62^c^	0.38^c^	0.35^c^	0.40^c^	0.03	**0.46**	0.85	0.00^d^	0.03^d^	−0.06^d^	0.12	−0.21	0.10
Drums	0.58^c^	0.29^c^	0.26^c^	0.29^c^	−0.08	0.55^c^	**0.64**	−0.03^d^	−0.06^d^	−0.04^d^	0.02^d^	0.01^d^	0.01^d^
FCR	−0.05	^c^0.04	−0.04	−0.03	−0.01	−0.04	−0.04	**0.18**	0.14	−0.26	−0.04^d^	−0.01^d^	−0.03^d^
L*	0.02	0.15	0.13	0.16^c^	0.21^c^	−0.03	0.06	0.05	**0.20**	−0.10	0.31	−0.47	0.10
a*	0.12	0.08	0.12	−0.16^c^	0.01	0.06	−0.11	−0.15^c^	−0.23^c^	**0.22**	0.17	−0.23	0.17
b*	0.09	0.11	0.10	0.13	0.09	0.06	0.01	−0.09	0.31^c^	0.27^c^	**0.23**	−0.24	0.24
pHu	−0.17^c^	−0.18^c^	−0.17^c^	−0.18^c^	−0.12	−0.11	−0.04	0.01	−0.19^c^	−0.32^c^	−0.19^c^	**0.34**	0.16
WS	0.12	0.05	0.06	−0.02	−0.02	0.04	−0.03	0.00	−0.05	0.19^c^	0.00	−0.14	**0.15**

a Standard error for heritability estimates ranged from 0.02–0.05.

b Standard error for the additive genetic correlation coefficients ranged from 0.00–0.12.

c Partial phenotypic correlation coefficients with a superscript represent coefficients that are significantly different from 0 (*p* < 0.05).

d Additive genetic correlation coefficients with a superscript represent cases where the associated SE is larger than the coefficient.

BW, body weight 2 days before slaughter (20–24w; kg); BrW = breast meat weight (kg); Fillets = Pectoralis major weight (kg); Tenders = Pectoralis minor weight (kg); BMY, breast meat yield (% BW); FCR, feed conversion ratio (kg/kg); pHu = ultimate pH; WS, white striping (0–3); L* = fillet lightness; a* = fillet redness; b* = fillet yellowness.

Heritability estimates for the trichromatic coordinates (L*, a*, and b*) were similar in magnitude, showing all three traits to be moderately heritable. The heritability estimates for L* (*h* ^2^ = 0.20 ± 0.02) and a* (*h* ^2^ = 0.22 ± 0.02) were similar to the previous estimates reported in turkeys. Estimates for L* and a* have been reported to range between 0.12 to 0.27 and 0.21 to 0.30, respectively (Le Bihan-Duval et al., 2003; Aslam et al., 2011). However, the estimate for b* (*h* ^2^ = 0.23 ± 0.02) was higher than previous estimates of 0.15 and 0.14 in turkeys (Le Bihan-Duval et al., 2003; Aslam et al., 2011). In comparison to estimates for breast meat colour in broilers, the heritability of L* from the present study was lower than the published range of 0.29–0.59 (Le Bihan-Duval et al., 2001, 2008; Gaya et al., 2006, 2011; Felício et al., 2013; Alnahhas et al., 2016). However, h^2^ estimates for a* and b* were within the published ranges of 0.21–0.57 and 0.12 to 0.55, respectively (Le Bihan-Duval et al., 2001, 2008; Gaya et al., 2006, 2011; Felício et al., 2013; Alnahhas et al., 2016). Similar to breast meat colour, only two estimates for pHu have been reported for turkeys. The heritability estimate for pHu (*h* ^2^ = 0.34 ± 0.05) in the present study was much greater than the previous estimates (*h* ^2^ = 0.09, Le Bihan-Duval et al., 2003; *h* ^2^ = 0.16, Aslam et al., 2011). Since *h* ^2^ is a population-specific parameter, there are several factors that can lead to the observed difference in *h* ^2^ estimates between these studies, including the genetic line used, use of purebred or commercial birds, or the sex of the birds. However, this estimate was similar to the majority of previously published estimates in broiler chickens reporting an average heritability of 0.34 (Le Bihan-Duval et al., 2001, 2008; Gaya et al., 2006; Gaya, et al., 2011; Felício et al., 2013; Alnahhas et al., 2016). Overall, these estimates suggest that there is a moderate genetic component to breast colour and pHu. The development of highly automated measurement techniques may allow for accurate measurements of these traits without sacrificing processing line speed. This would allow for the collection of many phenotypes necessary for genetic selection for improved meat quality.

To the best of our knowledge, this is the first published heritability estimate for WS in turkeys. In the population studied, WS was found to be moderately heritable (*h* ^2^ = 0.15 ± 0.02) suggesting the potential for genetic selection against WS in turkeys. This estimate was low compared to reported estimates for broiler chickens which ranged from 0.19 to 0.50 when using a similar 0–3 scoring system (Bailey et al., 2015; Lake et al., 2021) and 0.65 when using a 0–2 scoring system (Alnahhas et al., 2016). With this being the first estimate of heritability in turkeys, the only comparable estimates are those of broiler chickens. The goal of comparison is therefore not to arrive at the same estimate but express the potential difference between the species. Due to longer intense directional selection for growth and meat yield in broiler chickens and the well documented relationship between WS and growth, a larger genetic variation in chickens would not be surprising. The difference in heritability observed between these species could also suggest that WS is still in its infancy in turkeys resulting in the reduced genetic variance observed in the present study. If this is the case, that only emphasizes the importance of introducing genetic selection against WS in hopes to prevent further development of the myopathy.

### Genetic and Partial Phenotypic Correlations

Additive genetic and partial phenotypic correlation coefficients between the traits are presented in Table 2. As expected, the genetic and partial phenotypic correlations between the body and carcass component weights were favorable. The strongest correlations were observed between BW and BrW (*r*_*g*_ = 0.74 ± 0.02, *r*_*p*_ = 0.78), BW and thighs (*r*_*g*_ = 0.73 ± 0.04, *r*_*p*_ = 0.62), and BW and drums (*r*_*g*_ = 0.66 ± 0.04, *r*_*p*_ = 0.58). These are the three largest muscle groups which make up the largest portion of overall body weight. Moderate, favorable genetic and partial phenotypic correlations were observed between BrW and thighs (*r*_*g*_ = 0.33 ± 0.06, *r*_*p*_ = 0.38) and BrW and drums (*r*_*g*_ = 0.24 ± 0.06, *r*_*p*_ = 0.29), which is logical as birds with larger and heavier breast muscles require stronger leg muscles to support walking ability. However, since the genetic correlation estimates are not perfect, stronger emphasis placed on selection for breast traits may still lead to an unbalanced development of the leg muscles. Correlations between body and carcass component weights and FCR were all weak (−0.04 < *r*_*g*_ < 0.04), except for fillets (*r*_*g*_ = 0.16 ± 0.10, *r*_*p*_ = −0.04), suggesting a moderate, unfavorable relationship between fillet weight and FCR in the studied population. This was not the case in previously published studies in turkeys which showed a genetic correlation of BW and FCR ranging from 0.10 to 0.19 (Case et al., 2012a; Abdalla et al., 2019). All correlations between the studied traits and FCR were also weak (−0.04 < *r*_*g*_ < 0.04) with the exception of FCR and L* (*r*_*g*_ = 0.14 ± 0.10, *r*_*p*_ = 0.05) which showed a moderate, favorable genetic correlation and FCR and a* (*r*_*g*_ = −0.26 ± 0.09, *r*_*p*_ = −0.15) which showed moderate, favorable genetic and phenotypic correlations. This suggests that selection for FCR may affect meat quality, however, this may be due to the connection between FCR and fillet weight and should be investigated further.

Several correlations were observed in the present study that support the mechanism for the development of PSE meat. Moderate, unfavorable correlations were observed between BW and pHu (*r*_*g*_ = −0.18 ± 0.08, *r*_*p*_ = −0.17) as well as BrW and pHu (*r*_*g*_ = −0.19 ± 0.08, *r*_*p*_ = −0.18). The correlation between size of the bird and pHu of the breast muscle has been documented with larger, faster-growing lines showing a tendency to have a faster pH decline and lower pHu (Wang et al., 1999; Owens et al., 2002; Updike et al., 2005; Aslam et al., 2011). Another important relationship is between muscle temperature during the chilling process and colour of the final meat product. Rathgeber et al. (1999) showed that delayed chilling of turkey breasts led to an increase in L*, a*, and b*. Similarly, Mckee and Sams (1998) reported increases in L* when turkey breasts were held at 40°C for 2 h compared to breasts held at lower temperatures. A similar relationship was found in the current study with heavier BrW measurements showing an unfavorable genetic correlation with L* and b* (*r*_*g*_ = 0.43 ± 0.058 and *r*_*g*_ = 0.19 ± 0.062, respectively) potentially due to the increased chilling time required for larger carcasses resulting in higher carcass temperatures and functional protein degradation. Finally, the relationship between pHu and L* was strong and negative in the present study (*r*_*g*_ = −0.47 ± 0.09, *r*_*p*_ = −0.19) similar to previously published genetic correlation estimates in turkeys which ranged from −0.42 to −0.53 (Le Bihan-Duval et al., 2003; Aslam et al., 2011). Decreases in pH of the breast muscle lead to degradation of the functional proteins causing increased light refraction (increased L*) in the final meat product (Warriss and Brown, 1987; Barbut, 1993) as supported by our results. The strong genetic correlations between pHu and colour support the suggestions of Barbut (1997) for future use of non-destructive, easier to automate colour measurements over more invasive pH measurements in selection against PSE meat.

Several studies have reported the relationship between growth and muscle myopathies, including WS, in turkeys and broiler chickens (Mudalal et al., 2015; Russo et al., 2015; Baldi et al., 2018; Soglia et al., 2018; Carvalho et al., 2021). In the present study, a moderate, unfavorable genetic correlation was observed between WS and BW (*r*_*g*_ = 0.26 ± 0.07, *r*_*p*_ = 0.12) and WS and BrW (*r*_*g*_ = 0.25 ± 0.07, *r*_*p*_ = 0.05), suggesting that selection for growth will also lead to an increase in WS prevalence if additional traits are not used to balance correlated effects. WS also showed weak to moderate, unfavorable correlations with the studied meat quality traits, the strongest of which was with b* (*r*_*g*_ = 0.24 ± 0.08, *r*_*p*_ = 0.00). Moderate genetic correlations were also estimated with pHu (*r*_*g*_ = 0.16 ± 0.10, *r*_*p*_ = −0.14) and a* (*r*_*g*_ = 0.17 ± 0.09, *r*_*p*_ = 0.19), while a weak genetic correlation was estimated between WS and L* (*r*_*g*_ = 0.10 ± 0.09, *r*_*p*_ = −0.05). Varying results have been previously published regarding the relationship between WS and meat quality in poultry. Carvalho et al. (2021) reported that turkey breasts affected by WS showed increased L* and b* but no difference in pHu. In contrast, Soglia et al. (2018) reported no significant difference between WS severity and pH or breast meat colour in turkeys, while Mudalal (2019) reported a significant increase in b* and pHu in raw turkey breasts affected by WS. In broiler chickens, significant differences have been observed in various meat quality traits, such as pHu, L*, a*, and b*, between normal breast and breast affected by WS, however, with varying degrees of consistency (Kuttappan et al., 2013a; Petracci et al., 2013; Trocino et al., 2015; Baldi et al., 2018). Future research should focus on understanding the biological mechanism and relationship between WS and meat quality to better understand these inconsistent results. Development of machine vision algorithms to quantitatively score white striping would assist in increasing the amount and accuracy of data collection to support this research and provide a more robust trait for future selection programs.

## Conclusion

In the present study, we estimated the genetic parameters for white striping severity, breast meat colour, and pHu alongside several body and carcass component weights and FCR in turkeys. We have reported the first estimate of heritability for white striping in turkeys and have added to the body of knowledge surrounding heritability of meat quality. The estimates show potential for future genetic selection for improved meat quality and reduced white striping severity. The estimates also provide insight into the unfavorable effects of selection for growth, meat yield, and efficiency have on meat quality.

## Data Availability

The raw data supporting the conclusion of this article will be made available by the authors, without undue reservation.
